# The Potential for Undue Patient Exposure during the Use of Telementoring Technology

**DOI:** 10.7759/cureus.7594

**Published:** 2020-04-08

**Authors:** David P Darrow, Anthony Spano, Andrew Grande

**Affiliations:** 1 Neurosurgery, University of Minnesota, Minneapolis, USA; 2 Radiology, University of Minnesota, Minneapolis, USA

**Keywords:** medical education, hipaa, privacy, telementoring, video recording, surgery

## Abstract

Background

Surgical telementoring holds great promise for safe and effective patient care and medical education, but recording and streaming audio and video introduces the potential for exposure of patient information. Physicians maintain an ethical responsibility to protect the privacy of patients, and privacy violations may carry significant legal liability. Despite the legal treatment of violations as discrete, methods for quantifying and characterizing the exposure of patient information during procedural recordings are lacking. This study is the first to quantify the potential risk for violation of privacy when using a wearable, telementoring technology capable of video and audio recording during surgical procedures in various locations including the operating room, interventional radiology suite, and the intensive care unit.

Methods

A head-mounted recording device, Google Glass™, was used to record routine neurosurgical and critical care procedures in a convenience sample of patients. Periods of maximal risk, including the beginning of procedures, were targeted. Recordings were manually coded for discrete instances of exposure of directly identifying information and indirectly identifying information.

Results

Twenty-two procedures were recorded for a total of 12 hours, during which 807 directly identifiable exposures were found. The overall average rate of exposure was 1.13 exposures per minute. Most exposures were full-face images (90%), names (7%), or phone numbers (3%). Indirectly identifying exposures were found to be tattoos, genitals, and caretaker names. The rate of exposures was found to be lower in the operating room (OR) when compared to the intensive care unit (ICU) or interventional radiology (IR) suite (p = 0.0376).

Conclusions

High rates of potential privacy violations were discovered and found to be related the location of the procedure. Sterile draping of the face prior to recording, when appropriate, would mitigate most exposure risk, though patient names and unique tattoos may be an underappreciated source of potential exposure. This study establishes the most conservative baseline to compare techniques for preventing exposure of patient information on telementoring or video/audio recording/streaming platforms.

## Introduction

Intraoperative and perioperative video recording have become a mainstay practice for operative feedback and medical education. Analyses of these recordings provide insight into novel procedures and allow students to critically consider and correct their performance [1,2]. Advances in robotics, recording, and bandwidth technology have facilitated the implementation of telementoring technologies in medical education. Machinery and peripherals such as the da Vinci® allow remote teachers to engage fully with the operation and guide students using telestration in real time [3-6]. Telementoring technologies are continually becoming more affordable, and implementation can be as simple as putting on a pair of glasses [7,8].

The development of compact telementoring platforms, such as Google Glass™, provides students with tailored feedback and reduces procedural complication risk of teaching students, creating an ethical impetus for these high-fidelity technologies [9]. Telementoring technologies establish an active connection to the procedure, wherein a distant surgeon can guide a trainee as they learn a new procedure or encounter an unexpected emergency [6,10-12]. Telementoring could revise the “see one-do one-teach one” didactic standard, giving students a more refined understanding and an additional means to assess their skill and knowledge [11,13,14].

Telementoring may also benefit practicing physicians by providing a dynamic and effective method of teaching, even when trainers or trainees are at remote locations [9]. Medical education may be more effectively distributed to trainees while allowing attending surgeons to direct more of their time toward their practice [3,12,15]. Learning new surgical procedures is difficult and can be dangerous to patients. Surgeons who are less experienced have a higher frequency of complications [16]. With the use of telementoring, surgeons may be able to use the expertise of a more experienced surgeon in order to safely learn new techniques [10,17]. As a result, telementoring can extend medical education so that students and practicing physicians can receive advice from experts anywhere in the world [14,18]. Furthermore, telemonitoring can help developing nations train practitioners who would otherwise lack the necessary educational resources [7,19].

Telementoring may also empower patients to become knowledgeable consumers who can find expert and experienced care that would otherwise not be geographically accessible [3,4]. As a result, patients may be able to access previously unavailable, quality care. The addition of a mentoring surgeon comforts the patient and provides expert oversight during their procedure [10].

Although telementoring provides a unique and potentially effective platform for medical education, its integration may infringe on important ethical standards [20]. Effective implementation of telementoring requires striking a balance between security and accessibility [8]. Expanding connections to external networks provides a potential route for the loss of private data gathered during telementoring and dissuades patients from consenting to recording [21-24]. Transmission over unsecure, public networks could result in HIPAA violations by exposing identifiable patient information such as images of a patient’s face [25]. In order to understand the potential liability and provide an effective learning platform, it is necessary to determine the propensity for telementoring technology to violate HIPAA standards and patient privacy [4,6]. Such analysis will provide a foundation to create robust directives for liability and licensing for what stands to revolutionize medical education [10,26].

Rates of potential exposure during video recording for the purpose of telementoring or self-study have not been well described. To establish baseline rates and better understand patient privacy, this study attempts to characterize and establish the risk of exposing patient privacy in particularly vulnerable environments, namely around and during the time of surgery or procedures with the use of a point-of-view audio and video recording platform capable of telementoring.

## Materials and methods

A convenience sample of patients were recruited by the principal investigator during the course of routine neurosurgical procedures. The University of Minnesota Institutional Review Board approved this study, and the PI obtained consent for recordings directly from patients. All patients prospectively consented for the study. Recording was continuous and focused on the most exposed portions of longer operations, always including the beginning of procedures when patients were being prepared. Google Glass™ was worn until the procedure/operation ended, the extended battery was depleted, or the use of an intraoperative microscope was required, necessitating removal of the glasses. The videos were stored locally on the Google Glass™ hardware and later transferred to a secure and encrypted hard drive.

The types of exposures and associated risk were divided into several categories as follows. Identifying variables provide a direct identification of the patient (e.g., name or phone number) and carry the most severe risk of exposure. Intermediate risk quasi-identifiers are variables that help but are not sufficient to uniquely identify the patient (e.g., gender, DOB). Low risk non-identifying variables do not expose the patient to identification [27]. Non-identifying variables include lab values or potentially sensitive exposures (e.g., pictures of genitalia) [28]. The variables examined in this study were applied to both audio and video as well as quasi-identifiers examined in previous studies (Table 1) [27,28]. Accordingly, we grouped these categories into directly identifying and indirectly identifying categories, corresponding to high risk and low risk, respectively.

**Table 1:** Identifying, quasi-identifying and non-identifying variables

HIPAA Identifying Variables	Quasi-Identifiers	Non-Identifiers and Sensitive Variables
Name	Date of Birth (day, month, or year)	Labs
Address	Gender	Genitalia
Phone numbers	Initials	
Fax Number	City	
Electronic mail address	Region	
Social Security numbers	Postal code	
Medical record number	IP address	
Health plan beneficiary numbers	URLs	
Account numbers	Device identifiers and serial numbers	
Certificate/license numbers	Caretaker Identifying Information	
Vehicle Identification and serial numbers	Tattoos	
Biometric identifiers	Room number	
Full face photographic images	Visitor Information	

Video and audio streams were coded for potential HIPAA violations as well as any other potential sources of identity that were discovered. To quantify a discrete instance of potential privacy exposure, a single count was defined as an instance of exposure that was interrupted by a period of non-exposure. While HIPAA guidelines were used as the standard, we further identified variables that would jeopardize patient privacy if a video had been publicly released. Additionally, we examined the video and audio streams for information that was not readily discernible but possibly discernible with advanced analysis. In order to investigate the difference in rates of exposure between locations, a quasi-Poisson generalized linear model was used to correct for over dispersion of counted data. Differences were considered statistically significant when a p-value was found to be less than 0.05. The R software system was used for all statistical calculations [29]. Google Glass™ is not FDA approved for any medical use.

## Results

Nearly 12 hours of video and audio were recorded during 22 total procedures. There were 10 procedures recorded in the intensive care unit (ICU), five procedures in the interventional radiology (IR) suite, and seven procedures in the operating room (OR). In total, there were 807 exposures, resulting in an overall average exposure rate of 1.13 per minute, as shown in Table 2. Procedures recorded included arterial line placement, central venous catheter placement, shunt tap/CSF collection, external ventricular drain placement, percutaneous rhizotomy for trigeminal neuralgia, posterior spine surgery, and cranial procedures.

**Table 2:** Total recorded procedural videos and potential exposures

Video	Procedures (minutes)
ICU (total min)	10 (239.73)
IR (total min)	5 (122.03)
OR (total min)	7 (351.92)
Total Video	22 (713.68)
Total Number of Potential Exposures	807
Average Exposures per minute	1.13

Direct identifying information such as name, phone number, or full face photographs coded from the video and audio is listed in Table 3.

**Table 3:** Direct identifying variables

Identifying Variables: directly identify individual	Video	Audio
Names	57	48
Electronic Mail Address	0	0
Phone Numbers	21	0
Social Security Numbers	0	0
Home address	0	0
Medical Record number	0	0
Medical card number	0	1
Account numbers	0	0
Certificate/license numbers	0	0
Vehicle identifiers and serial numbers	0	0
Full face photographic images	729	0

Indirect identifying information such as date of birth or other uniquely discovered identifying information such as tattoos or even phone lock code coded from audio and video are listed in Table 4.

**Table 4:** Indirect identifying variables

Indirect Identifying	Video	Audio
Date of Birth	2	0
Date of Birth (Month and Year)	0	1
Year of Birth	0	0
Gender	0	1
Postal Code	0	0
Forward sortation area	0	0
City	0	0
Region	0	0
Initials	0	0
Fax Number	0	0
Geographical Information	0	0
Health plan beneficiary numbers	0	0
Web Universal Resource Locators	0	0
Internet Protocol Numbers	0	0
Biometric identifiers	0	0
Other: Tattoo	70	0
Other: Caretaker name	13	0
Other: Genitals	43	0
Other: phone lock code	0	1

We additionally compared the exposure rates for video between the different locations recorded, as listed in Table 5. The OR was compared to the ICU and IR suite, where a statistically significant difference was found between rates (p = 0.0376).

**Table 5:** Rates of exposure by location

Location	ICU	IR	OR
Average Exposure/min	1.552	2.385	0.409

## Discussion

To our knowledge, this is the first study to quantitatively characterize the risk of potential privacy exposure and HIPAA violations in video and audio recordings of operations and procedures during the routine use of telementoring technology. As a necessary component of a telementoring platform, recorded or streaming video and audio may pose significant risk primarily through exposure of a patient’s face, which was an order of magnitude more common than the second most common exposure in this study, a patient’s name.

Our hope is to quantify and characterize exposure rates for future comparison in studies aimed at reducing these rates from what we consider to maximally exposed situations where no special precautions were taken to mitigate exposure risk. This approach was chosen to provide frank, naïve estimates for the risk of exposure to later compare with informed, prevention strategies.

Overall, we found an average rate of more than one exposure per minute. Importantly, full-face photographs composed more than 90% of the Direct Identifying exposures, while names (7%) and phone numbers (3%) composed the rest. Exposures during audio recordings were dominated by names (98%) and a medical record number (2%). Preventing the recording of faces by waiting to begin recording until after complete draping, when appropriate, would significantly reduce the exposure risk.

Indirect Identifying categories were dominated by novel categories discovered during recordings as potential privacy exposures and included tattoos (55%), genitals (34%), and a caretaker name (10%). There were only rare exposures of birth date (1%) on audio or video. Unique tattoos were an unexpected driver of potential exposures and would also be minimized by postponing video recording until after sterile draping.

Our approach to analysis of the video was based on the assumption that a video could be released to the general public in a retained file. If a video was released via a public network, then the video file would be reviewable without the limitation of traditional broadcast videos. Sophisticated analysis tools may increase the probability of revealing subtle information about a patient. Accordingly, we re-examined our own data to further code for images containing variables that, while not readily discernible, hold the potential for exposure if sophisticated techniques were used. We identified 25 more instances of possible name exposure and five more instances of full-face exposure. Only three discrete instances of potential name exposure were during procedures when the name was not already discernible, and all five potential full-face exposures were during videos when the face had already been discernible. In general, it is important to recognize that today, the release of a video implies that the entire file can be scrutinized using advanced video analysis tools, and even the metadata (embedded data) of the video file can reveal information about the patient or procedure.

Given these results, the periods of greatest potential for exposure include the beginning and end of a procedure or operation, since any identifiable information is usually covered by sterile draping. We intentionally chose to record video as early as possible to examine this period of maximal risk in this first study. Our data revealed that location may play a role in the rate of exposure, which corresponds with common sense about the level of control in a specific environment. When we compared the rates of exposure in the operating room with rates of exposure in the interventional radiology suite (which also involved procedures where the face is exposed) and in the intensive care unit, we discovered that exposure rates in the operating room were statistically lower, despite heterogeneous data. Longer procedure times and a more controlled environment readily explain this difference.

Limitations

We acknowledge several limitations of our study. While we attempted to include a variety of locations and procedures with potentially high exposure rates, we only included neurosurgical and critical care procedures. Our length of recording time was limited to a couple of hours, so only the beginning of some of the longer operations was recorded, though we estimate much lower risk for exposure during the remainder of these cases and similar or less exposure risk at the end of procedures when compared with the beginning. Our methodology included only the use of a glasses-mounted platform, Google Glass™, while several other options exist with varying resolution. Our method of quantifying potential exposures attempts to describe discrete segments of potential violation, but there are many ways to measure exposure such as number of frames or seconds. We chose discrete instances because of the ability for indefinite review if released to the public digitally and the potential legal treatment. Further consideration should be given to other platforms that utilize different video resolution, frame rate, or encoding. Additionally, advanced analytical techniques for recovery could also be explored on control data.

## Conclusions

Video and audio recording during procedures or operations using a head-mounted camera may pose substantial risk for exposure of patient-identifiable information when no significant prevention strategies are employed. While the majority of risk stems from the exposure of the faces of patients where mitigation is easily achieved (draping), some risk remains from patient names posted on the surroundings. Controlled environments where patients are more uniformly draped (OR) may carry reduced risk when compared to the ICU or IR suite. Strategies for reducing or removing exposure risk on audio and video recordings may assess efficacy against the results of this study.
